# Supplementary material for: P32-specific CAR T cells with dual antitumor and antiangiogenic therapeutic potential in gliomas
Source: Nat Commun. 2021 Jun 14;12:3615. doi: 10.1038/s41467-021-23817-2 (PMC8203650; doi:10.1038/s41467-021-23817-2)
Supplement: Supplementary file 1 — Supplementary figures [file 41467_2021_23817_MOESM1_ESM.pdf]

Supplementary figure 1

a

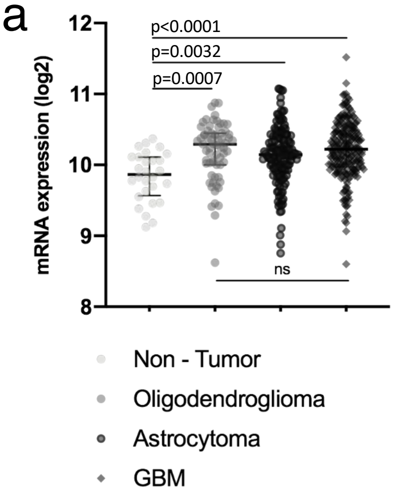

	Sample count	Median (log2)
Non-Tumor	28	9.87
Oligodendro.	67	10.29
Astrocytoma	147	10.14
GBM	219	10.22

b

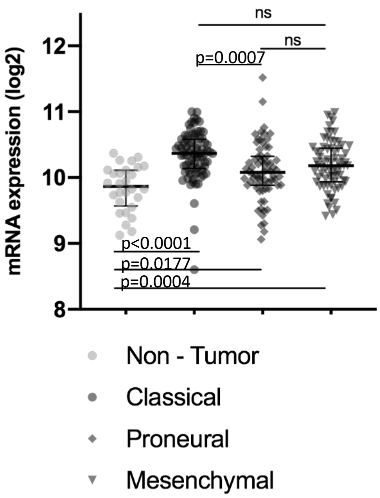

	Sample count	Median (log2)
Non-Tumor	28	9.87
Classical	79	10.37
Proneural	70	10.08
Mesenchymal	70	10.18

c

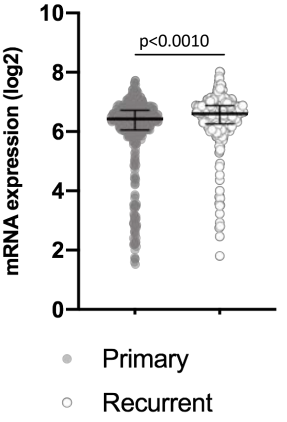

	Sample count	Median (log2)
Primary	651	6.42
Recurrent	333	6.60

d

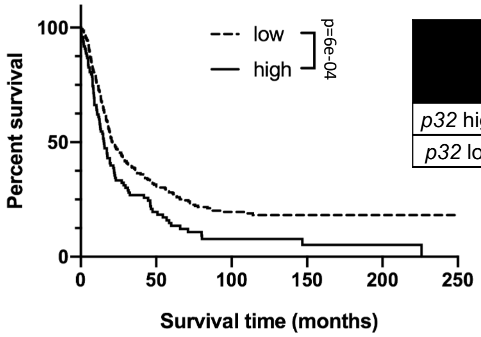

	Sample count	Median Survival (months)
p32 high	189	16.8
p32 low	333	25.5

e

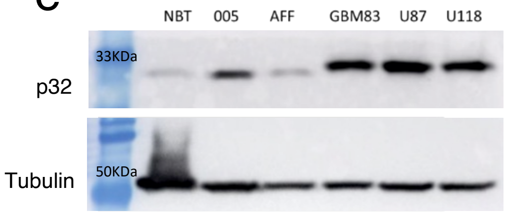

Supplementary Fig 1. Analysis of p32 expression levels in murine and human glioma samples.
a *p32* mRNA expression from Rembrandt database comparing non tumor tissue with samples belonging to different glioma histologic types. **b** *p32* mRNA expression among samples belonging to three GBM molecular subtypes (Rembrandt dataset). **c** *p32* mRNA expression from CGGA database comparing primary brain tumors with recurrent ones. Statistical significance was determined by one-way ANOVA, Dunnett′s multiple comparisons test. Median with interquartile range is shown in each graph. Each dot in the graphs represents a patient sample. *N* (sample count) for **a-c** appears in the table below each graph. **d** Kaplan-Meir curves comparing survival times between low and high *p32* expression in glioma patients (Rembrandt dataset). Cutoff criteria applied based on median *p32* expression for total patient cohort (10.19). *N* (sample count) appears in the table next to the graph. Log-rank test was used for statistics. Datasets were downloaded from http://gliovis.bioinfo.cnio.es/ **e** Protein level of p32 evaluated by western blot. Samples included mouse normal brain tissue (NBT), mouse 005 GSC, mouse differentiated glioma cell line AFFR53 (AFF), patient derived GBM83 GSC, and human glioma cell lines U87 and U118. Tubulin was used as loading control. Representative image of three independent experiments. Source data are provided as a Source Data file.

Supplementary figure 2

a

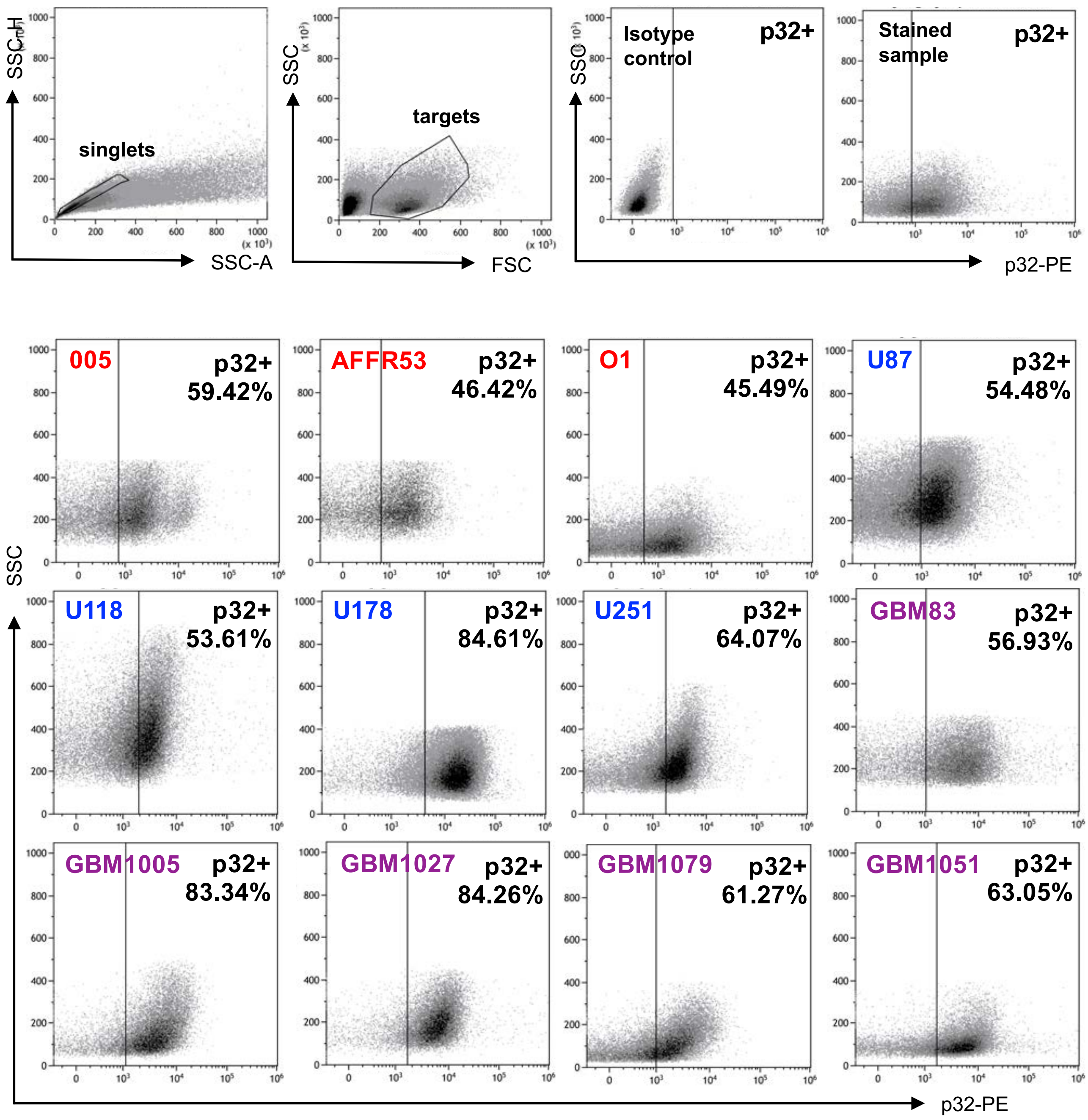

b

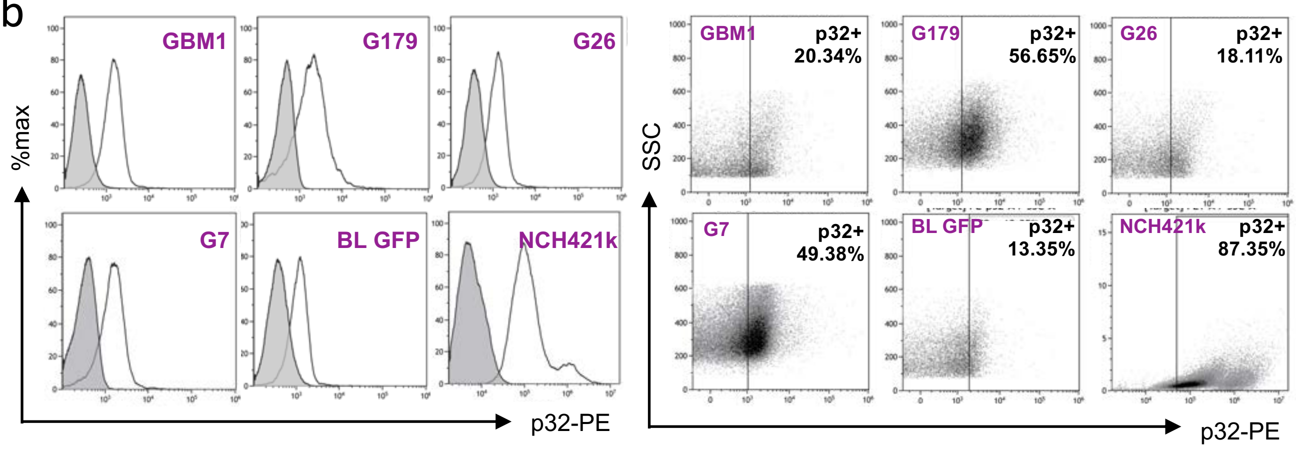

Supplementary Fig 2. Analysis of p32 surface expression levels in murine and human glioma samples. **a** p32 cell surface expression analyzed by FACS in murine glioma cells (005, AFFR53, O1), human cell lines (U87, U118, U178, U251) and patient derived mesenchymal (GBM83, GBM1005, GBM1027) and proneural (GBM1079, GBM1051) GSCs, percentages of p32+ cells are shown. **b** p32 cell surface expression analyzed by FACS of additional patient-derived GSCs, percentages of p32+ cells are shown. Each histogram and dot plot is representative of one of three independent experiments. Refer to Supplementary Table 1 for description of glioma cells.

Supplementary figure 3

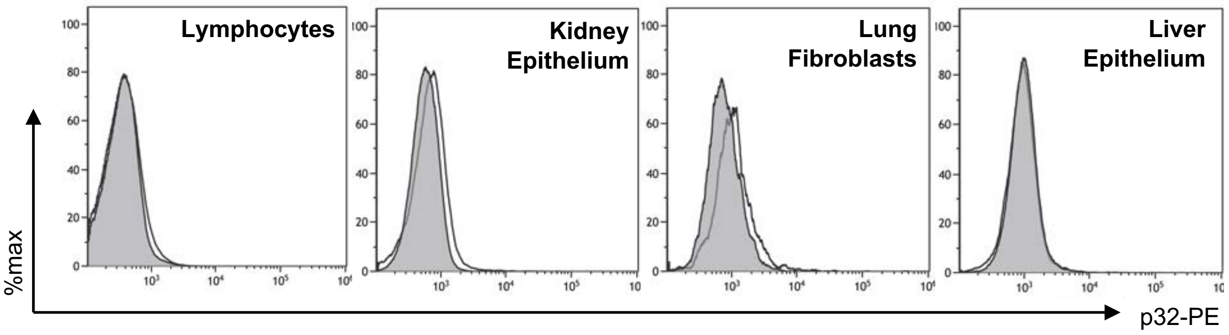

Supplementary Fig 3. Analysis of p32 surface expression levels in the indicated primary human cells by FACS. Each histogram is representative of one of three independent experiments. Refer to Supplementary Table 1 for description/source of primary human cells.

Supplementary figure 4

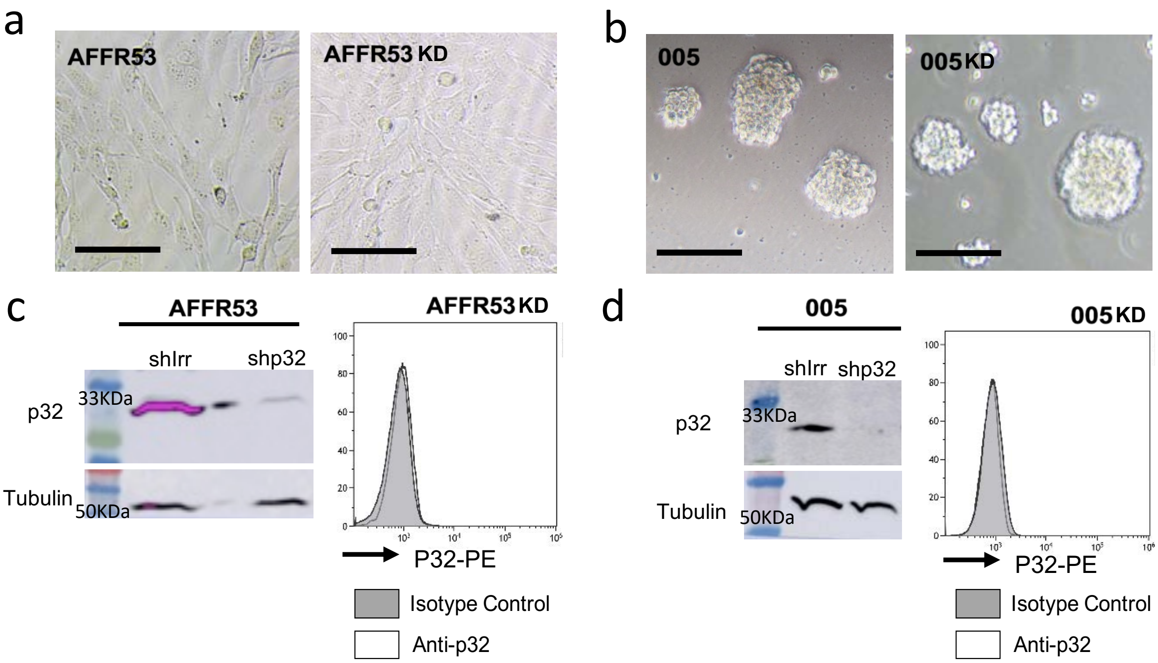

Supplementary Fig 4. Knockdown of p32 in murine glioma cells. (**a**-**b**) Bright field images (representative of three independent experiments) of the indicated murine glioma cell lines. Scale bar = 100 um. (**c**-**d**) AFF53 and 005 glioma cells were transduced with lentiviral vectors expressing shRNAs targeting either p32 (shp32) or control irrelevant target (shIrr). P32 knockdown was assessed by WB analysis, magenta indicates overexposure. Tubulin was used as loading control. Knockdown was also validated by surface expression of p32 using flow cytometry (histogram graphs on the right side). Each histogram and image is representative of three independent experiments. Source data are provided as a Source Data file.

Supplementary figure 5

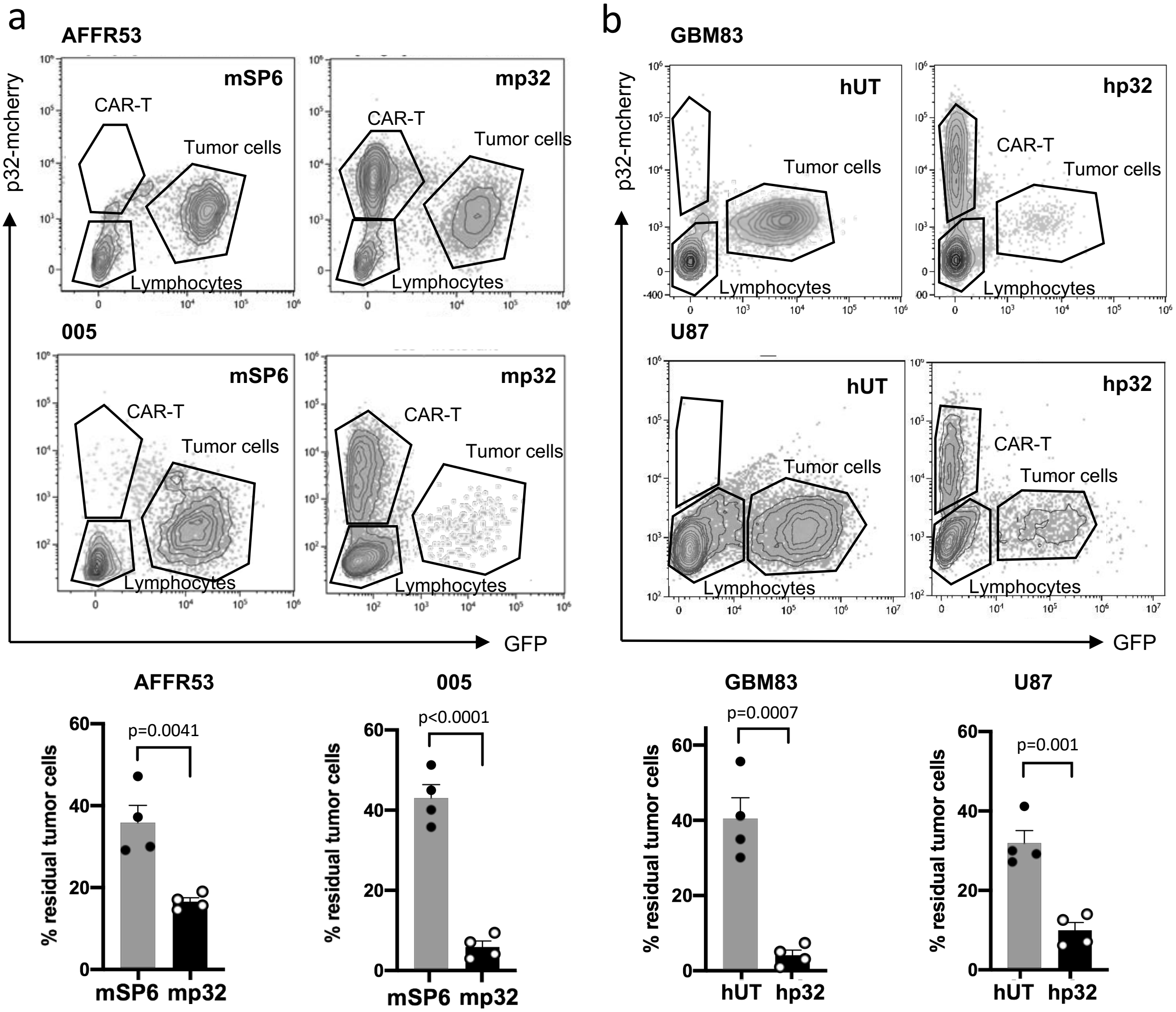

Supplementary Fig 5. Cytotoxic effect of murine and human p32 CAR T cells. **a** Cytotoxic action exerted by mouse CAR T cells against AFFR53 and 005 glioma cells was measured by the amount of residual GFP+ tumor cells after coculture (E:T=3:1) with p32 mCAR T (mp32) cells or a non-relevant mCAR T (mSp6) for 72 hours. Following incubation the amount of CAR T (mcherry+) and glioma cells (GFP positive) were examined via FACS. Graphs below the representative FACS plots show quantification of 4 independent experiments. **b** Same experiment as described in **a** using human CAR T cells and human GBM83 and U87 glioma cells. Graphs represent quantification of 4 independent experiments (two different donors). Data is presented as mean ± SEM and each dot represents the average for an independent experiment. Unpaired t test was used for statistical analysis. Two-tailed P value is shown Source data are provided as a Source Data file.

Supplementary figure 6

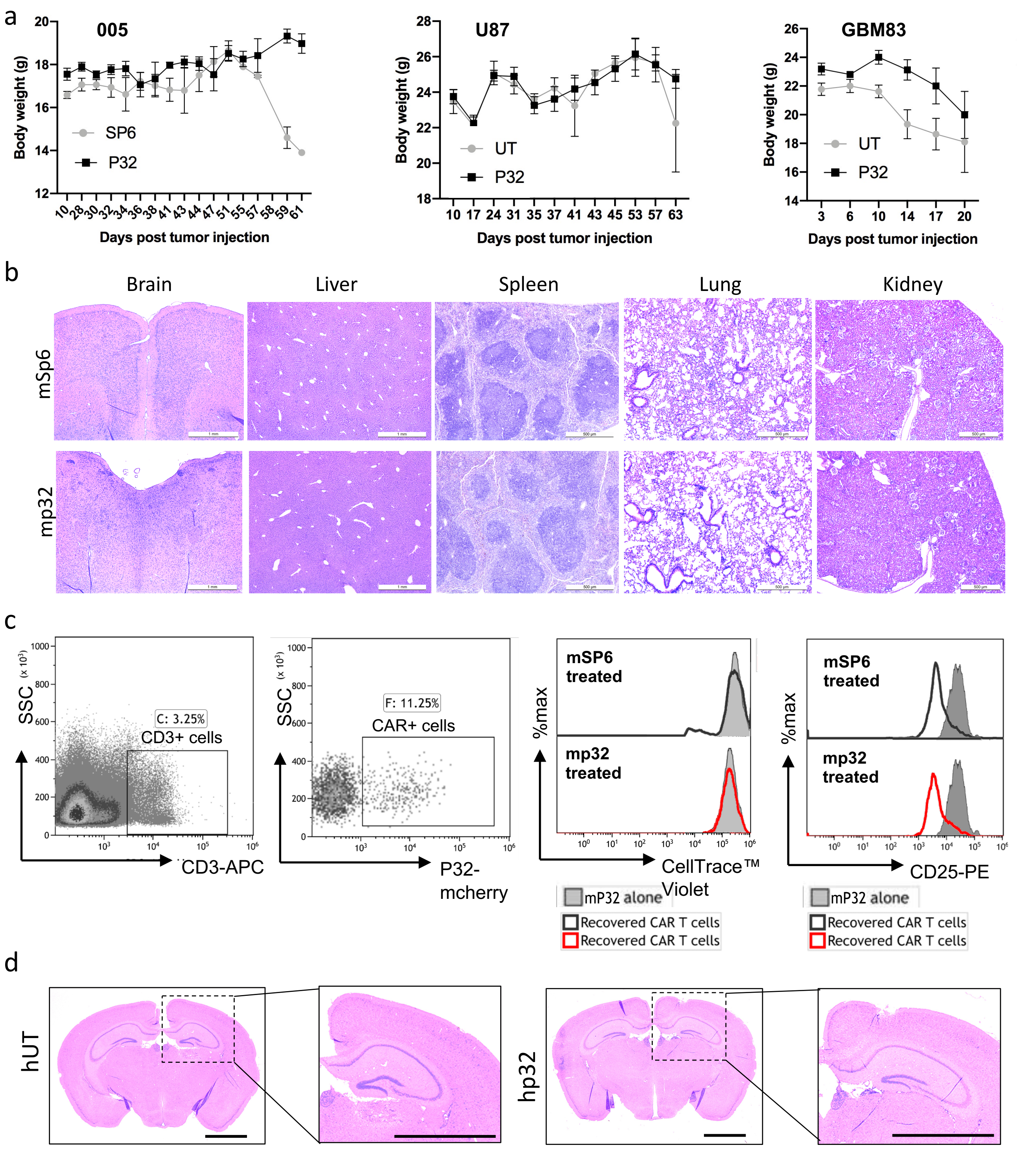

Supplementary Fig 6. No toxic adverse effect in CAR T injected mice. **a** CAR T administration (started 3/10 days post tumor induction) had no effect on the body weight prior to symptomatic period. Statistical analysis was performed using 2-way ANOVA to evaluate differences in weight in the GBM-asymptomatic period of time, data is presented as mean ± SEM. For 005: N= 7 mice SP6 group and N=8 in p32 group; For U87: N=6 mice for UT and 6 for p32; For GBM83: N=8 for each group. **b-c** C57BL/6 mice were pre-conditioned and 24 hrs later i.v. injected with either SP6 or p32 CAR T cells($1x10^7$ total T cells/mouse). **b** Pathology of the indicated organs was evaluated by H&E staining 15 days after lymphocytes injections. Representative images of *N*= 3 mice per group are shown. **c** Previously CellTrace-stained-T-cells were recovered from mice lung after 48hs. Proliferation and activation markers were analyzed in the recovered CAR-T cells Representative images and plots are shown, *N*= 4 mice per group. **d** Nude mice were stereotaxically injected with either untransduced (hUT) or p32 hCAR (hp32)T cells using the same coordinates as the intratumor and ICV injections in the treatment experiment ($1x10^7$ total T cells/mouse). Scale bar = 2 mm. Representative images of *N*= 3 mice per group are shown. Source data are provided as a Source Data file.

Supplementary figure 7

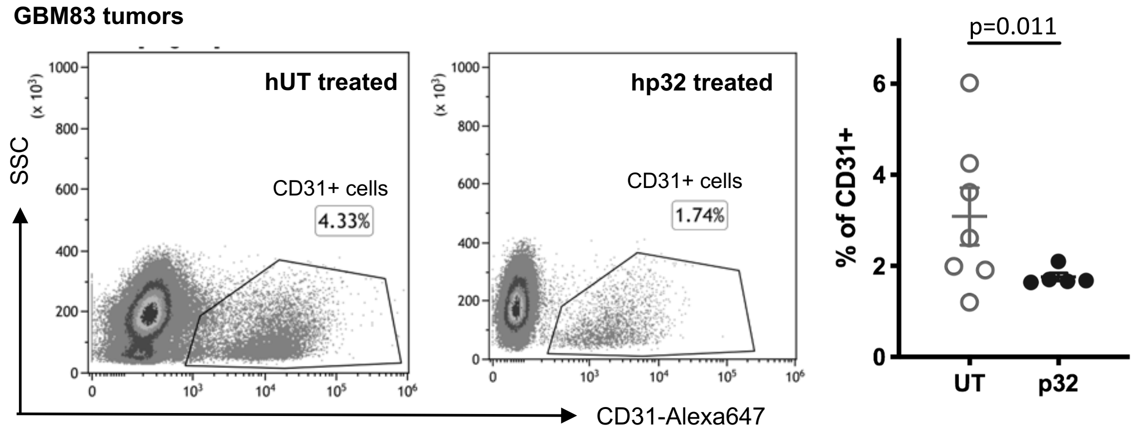

Supplementary Fig 7. GBM83 tumors were dissociated close to the experiment endpoint. After CD45+ cells depletion, the remaining fraction was stained for CD31 and the percentage of endothelial cells in the tumor fraction was analyzed by FACS. Data is shown as mean ±SEM. Each dot shown in the graph represents individual values for a mouse (*N*= 7 for UT and *N*=5 for p32*).* Unpaired t test was used and two-tailed P value is shown. Source data are provided as a Source Data file.

Supplementary figure 8

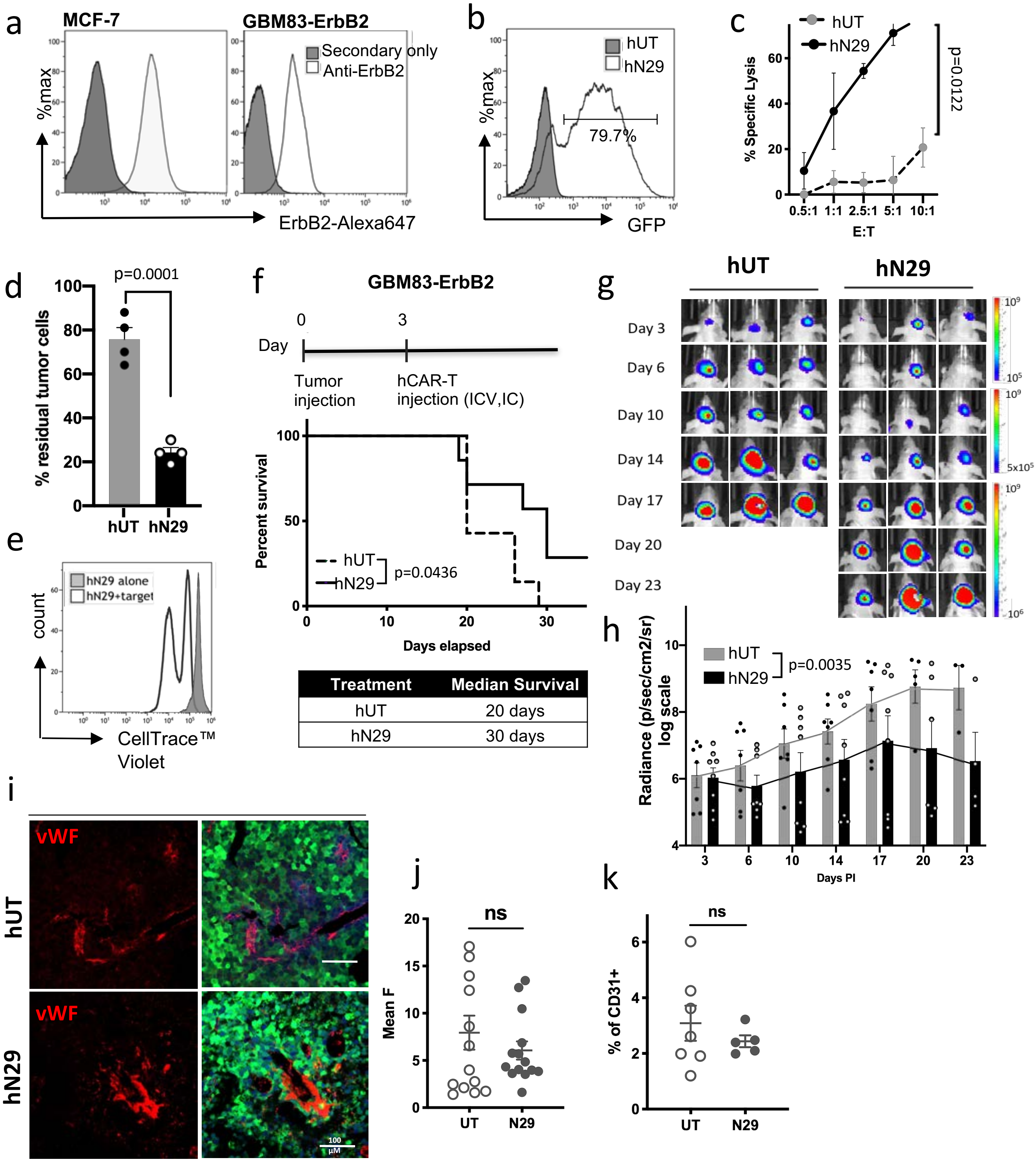

Treatment	Median Survival
hUT	20 days
hN29	30 days

Supplementary Fig 8. **a** GBM83 cells were engineered to overexpress the ErbB2 antigen. ErbB2 is recognized by the N29 antibody. **b** hN29 CART-cells were produced in order to recognize and kill ErbB2+ cells. Transduction efficiency was determined by GFP+ expression. Each histogram is representative of one of three independent experiments. **c-d** N29 CART-cells efficiently recognize and kill GBM83-ErbB2 cells. Data is shown as mean ±SEM **e** Proliferation of CAR-T+ cells was also evidenced in culture. Data represent mean±SEM. *N*=4 independent experiments. Unpaired t test (with Welch's correction for **c**) was used for statistical analysis. Two-tailed P value is shown. **f** In a similar fashion that hp32 CAR-T cells, treatment with hN29 CAR-T cells extended median survival of mice bearing ErbB2-GBM83 tumors *(N*=6 for hUT and N=7 for hN29). Two-sided Mantel-Cox (log-rank) test was used to address differences in median survival **g-h** Tumor size was monitored by IVIS, showing a shrinkage after N29 CAR-T treatment. Data represent mean±SEM, each dot represent one mouse, N=7 for hUT and N=8 for hN29. Two-way ANOVA was used to analyze overall differences in both groups towards the course of the experiment. **i-k** No anti-angiogenic effect was observed at endpoint in tumors analysis by either vWF staining of fixed tissue **i-j** or CD31 staining of dissociated tumors and FACS analysis (**k**). in **j** Data is shown as mean ±SEM. Each dot shown in the graph represents average of 3 measurements done per slide. A total of 13 slides originated in 4 different mice were stained per group. in **k** data is shown as mean ±SEM. Each dot shown in the graph represents individual values for a mouse (*N*= 7 for hUT and *N*=5 for hN29*).* For **j** and **k** unpaired t test was used and two-tailed P value is shown. Source data are provided as a Source Data file.

Supplementary figure 9

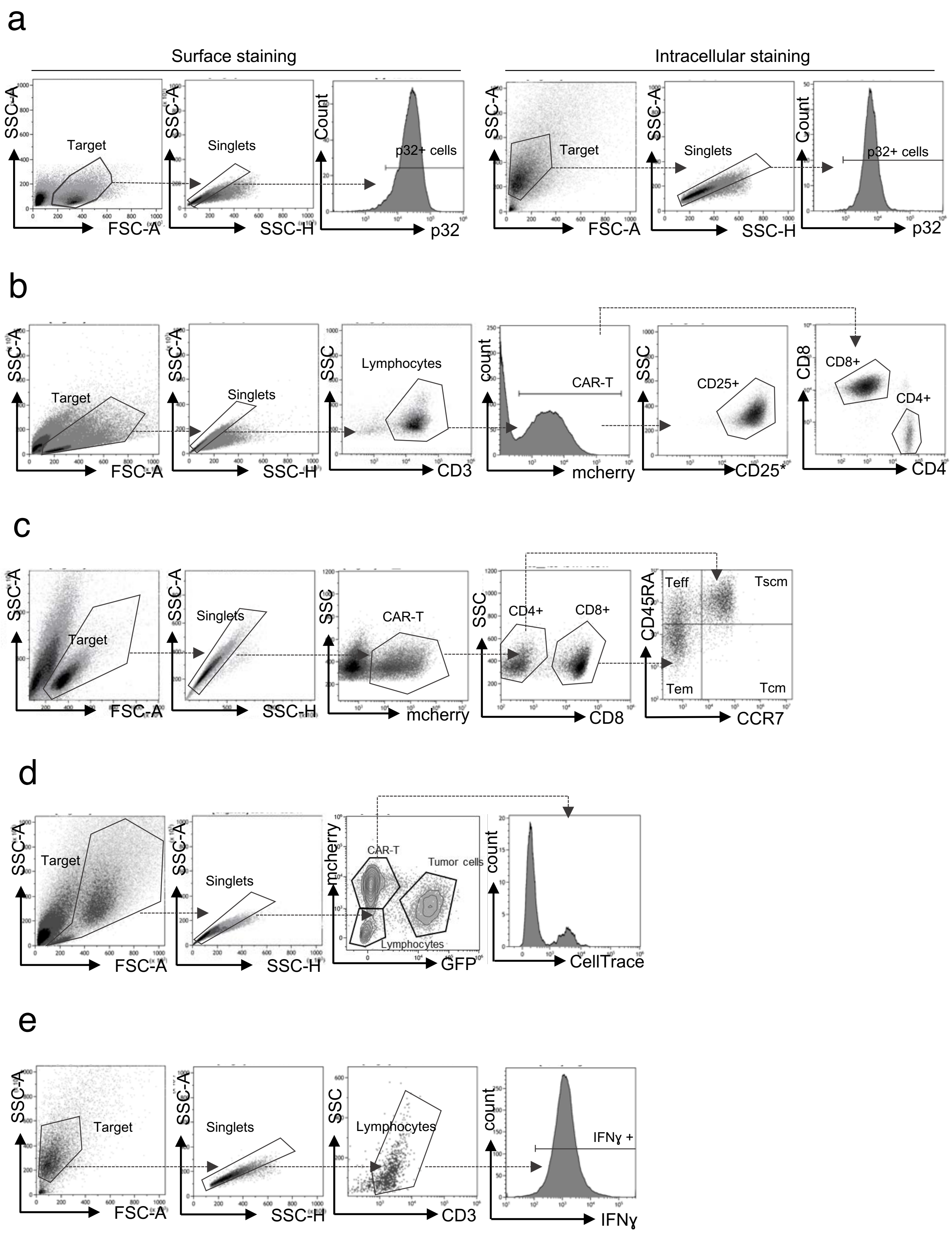

f

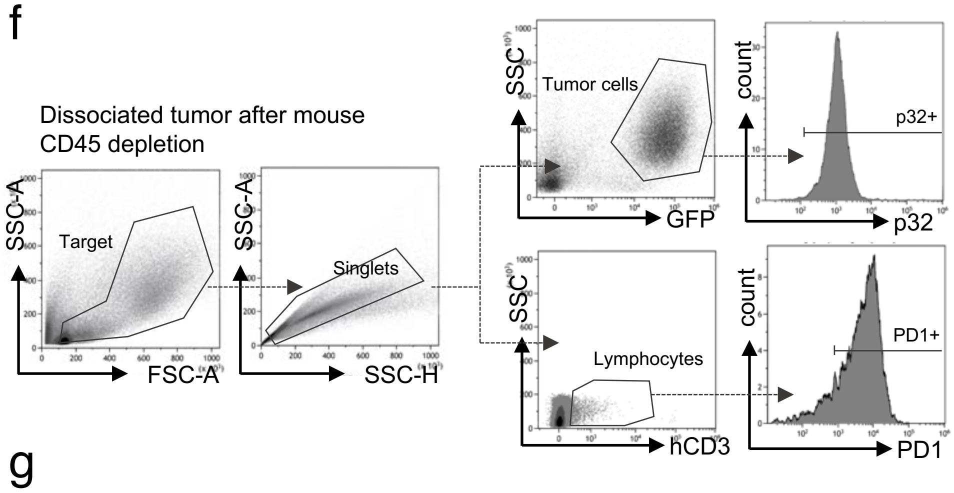

g

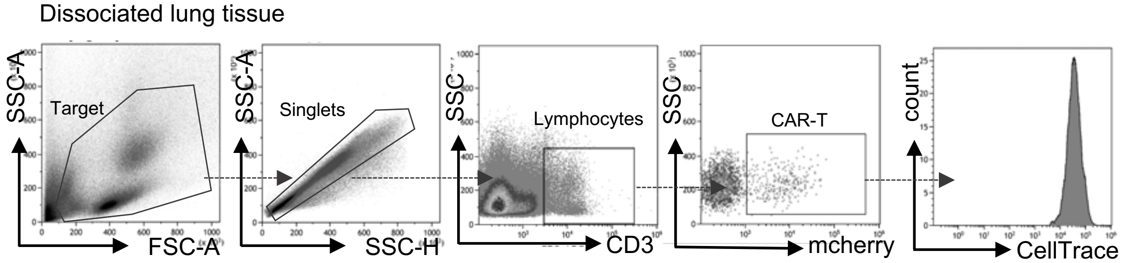

h

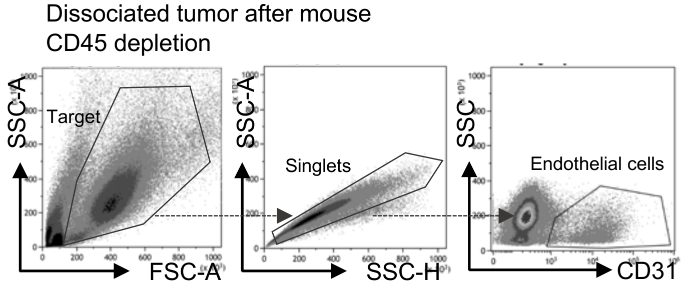

Supplementary Fig 9. Gating strategies used throughout the paper. **a.** Gating strategy used for p32 expression analysis as presented in Fig.1 c-d and Supplementary Fig. 2a-b, 3, 4c-d, 8a. Left panel represents an example of surface staining while the right panel shows the gating used for intracellular p32-staining. **b.** Gating used for p32-CAR-T (CD3+mcherry+) characterization, as shown throughout Fig. 2 and Supplementary Fig. 8b. for the N29-CAR-T (CD3+GFP+). Staining with CD25 marker is shown as an example. **c**. Gating used for phenotypic analysis of human CAR T cells presented in Fig. 2j. **d**. Strategy used for cytotoxicity (Supplementary Fig. 5, 8d) and proliferation assays (Fig 3b,f and Supplementary Fig. 8e). **e**. Gating strategy for intracellular IFNγ as presented in Fig. 3c. **f**. Gating strategy for analysis of dissociated GBM83-tumors after hp32-CAR-T treatment. Levels of p32 in tumor cells (GFP+) are shown in Fig. 4g and PD-1 levels in recovered T-Cells (CD3+) are shown in Fig. 4h. **g**. Strategy used to analyze dissociated lung tissue after CAR-T administration. CAR-T (CD3+mcherry+) proliferation levels are shown in Supplementary Fig. 6c. **h**. Gates used to measure amount of endothelial cells (CD31+) in tumors, as presented in Supplementary Fig. 7 and 8k.

Supplementary Table 1 – List of cell lines

Name	Human/ mouse	Description	Source
**005**	mouse	Murine glioma stem cells	Derived from pTomo-HRas-shp53 lentiviral induced tumor [1].
**AFF R53**	mouse	Murine glioma stem cells	Primary astrocytes transformed *in vitro* with pTomo-HRas-shp53 without fluorescence reporter [2].
**O1**	mouse	Pediatric glioma cell line	Derived from pTomo-FGFR1mut-shp53 lentiviral induced tumor.
**U87**	human	Human glioma cell line	ATTC (HTB-14)
**U118**	human	Human glioma cell line	ATTC (HTB-15)
**U178**	human	Human glioma cell line	ATTC (HTB-16)
**U251**	human	Human glioblastoma astrocytoma	Collection of Authenticated Cell Cultures (formerly known as U373MG), ECACC 09063001
**GBM83**	human	Patient-derived GBM cell lines	MES (Prof. Ichiro Nakano) [3]
**GBM1005**	human	Patient-derived GBM cell line	MES (Prof. Ichiro Nakano)
**GBM1027**	human	Patient-derived GBM cell line	PN (Prof. Ichiro Nakano) [4]
**GBM1051**	human	Patient-derived GBM cell line	PN (Prof. Ichiro Nakano) [4]
**GBM1079**	human	Patient-derived GBM cell line	PN (Prof. Ichiro Nakano) [4]
**G7**	human	Patient-derived GBM cell line	(Prof. SM Pollard) [5]
**G179**	human	Patient-derived GBM cell line	(Prof. SM Pollard/Peter Dirks) [5]
**G26**	human	Patient-derived GBM cell line	(Prof. SM Pollard) [5]
**BL**	human	Patient-derived GBM cell line	Prof. Santosh Kesari
**NCH421K**	human	Patient-derived GBM cell line	(Prof. C. Herold-Mende) [6]
**H-6067**	human	Primary dermal fibroblast	Cell Biologics
**H-6034**	human	Primary kidney epithelial cells	Cell Biologics
**H-6013**	human	Primary lung fibroblast	Cell Biologics
**H-6044**	human	Primary liver epithelial cells	Cell Biologics
**N7805-100**	human	Primary astrocytes	Gibco

Supplementary References:

1. Marumoto T*, et al.* Development of a novel mouse glioma model using lentiviral vectors. *Nat Med* **15**, 110-116 (2009).
2. Friedmann-Morvinski D, Bhargava V, Gupta S, Verma IM, Subramaniam S. Identification of therapeutic targets for glioblastoma by network analysis. Oncogene, (2015).
3. Mao P, *et al*. Mesenchymal glioma stem cells are maintained by activated glycolytic metabolism involving aldehyde dehydrogenase 1A3. Proc Natl Acad Sci U S A 110, 8644-8649 (2013).
4. Pavlyukov M, et al. Apoptotic Cell-Derived Extracellular Vesicles Promote Malignancy of Glioblastoma Via Intercellular Transfer of Splicing Factors. Cancer Cell 34, 119-135 (2018).
5. Stricker SH, et al. Widespread resetting of DNA methylation in glioblastoma-initiating cells suppresses malignant cellular behavior in a lineage-dependent manner. Genes Dev 27, 654-669 (2013).
6. Campos B, et al. Differentiation therapy exerts antitumor effects on stem-like glioma cells. Clin Cancer Res 16, 2715-28 (2010).
