# Supplementary material for: P32-specific CAR T cells with dual antitumor and antiangiogenic therapeutic potential in gliomas
Source: Nat Commun. 2021 Jun 14;12:3615. doi: 10.1038/s41467-021-23817-2 (PMC8203650; doi:10.1038/s41467-021-23817-2)
Supplement: Supplementary file 3 — Reporting Summary [file 41467_2021_23817_MOESM3_ESM.pdf]

nature research

Corresponding author(s): Dinorah Friedmann-Morvinski

Last updated by author(s): May 5, 2021

Reporting Summary

Nature Research wishes to improve the reproducibility of the work that we publish. This form provides structure for consistency and transparency in reporting. For further information on Nature Research policies, see our Editorial Policies and the Editorial Policy Checklist.

Statistics

For all statistical analyses, confirm that the following items are present in the figure legend, table legend, main text, or Methods section.

n/a	Confirmed	
☐	☒	The exact sample size (n) for each experimental group/condition, given as a discrete number and unit of measurement
☐	☒	A statement on whether measurements were taken from distinct samples or whether the same sample was measured repeatedly
☐	☒	The statistical test(s) used AND whether they are one- or two-sided<br>*Only common tests should be described solely by name; describe more complex techniques in the Methods section.*
☒	☐	A description of all covariates tested
☒	☐	A description of any assumptions or corrections, such as tests of normality and adjustment for multiple comparisons
☐	☒	A full description of the statistical parameters including central tendency (e.g. means) or other basic estimates (e.g. regression coefficient) AND variation (e.g. standard deviation) or associated estimates of uncertainty (e.g. confidence intervals)
☐	☒	For null hypothesis testing, the test statistic (e.g. F, t, r) with confidence intervals, effect sizes, degrees of freedom and P value noted<br>*Give P values as exact values whenever suitable.*
☒	☐	For Bayesian analysis, information on the choice of priors and Markov chain Monte Carlo settings
☒	☐	For hierarchical and complex designs, identification of the appropriate level for tests and full reporting of outcomes
☒	☐	Estimates of effect sizes (e.g. Cohen's d, Pearson's r), indicating how they were calculated

Our web collection on statistics for biologists contains articles on many of the points above.

Software and code

Policy information about availability of computer code

Data collection	For flow cytometry, Attune Nxt, for immunofluorescence data collection ZEN 2.3 software
Data analysis	We analyzed data using GlioVis (http://gliovis.bioinfo.cnio.es), GraphPad Prism 8, FIJI/ImageJ 2.0.0, Kaluza 2.1, Living Image software 4.7.3

For manuscripts utilizing custom algorithms or software that are central to the research but not yet described in published literature, software must be made available to editors and reviewers. We strongly encourage code deposition in a community repository (e.g. GitHub). See the Nature Research guidelines for submitting code & software for further information.

Data

Policy information about availability of data

All manuscripts must include a data availability statement. This statement should provide the following information, where applicable:

- Accession codes, unique identifiers, or web links for publicly available datasets
- A list of figures that have associated raw data
- A description of any restrictions on data availability

The glioma patient sequencing data analyzed in this study are available and were downloaded from http://gliovis.bioinfo.cnio.es (Rembrandt adult dataset was selected for tumors from different histological types and GBM molecular subtypes, and CGGA adult dataset to compare primary and recurrent tumors). The remaining data are within the paper and its supplementary information and Source Data files.

Field-specific reporting

Please select the one below that is the best fit for your research. If you are not sure, read the appropriate sections before making your selection.

- [x] Life sciences
- [ ] Behavioural & social sciences
- [ ] Ecological, evolutionary & environmental sciences

For a reference copy of the document with all sections, see nature.com/documents/nr-reporting-summary-flat.pdf

Life sciences study design

All studies must disclose on these points even when the disclosure is negative.

Sample size	No statistical method was used to pre-determine sample size, but the sample size was chosen to be adequate to receive significant results as determined by preliminary experiments.
Data exclusions	Mice that died for unknown reasons (no tumor lesions were detected) were excluded from analysis.
Replication	All experiments were repeated independently at least two times. Each individual experiment contained 2 or more biological/technical repeats. All the replication attempts were successful as described in the figures.
Randomization	Mice were randomly assigned to either control or treatment group. Male and female mice were used and age-matched. For in vitro assays, glioma cells were subcultured from the same initial plate and divided randomly between the plates or wells for subsequent co-culture with either control or CAR specific T cells.
Blinding	Since most of the experiments were performed and analyzed by the same person, blinding was not possible in most of the experiments. All imaging samples were quantified using the least biased approach possible. This included the use of image analysis programs as indicated in the methods section.

Reporting for specific materials, systems and methods

We require information from authors about some types of materials, experimental systems and methods used in many studies. Here, indicate whether each material, system or method listed is relevant to your study. If you are not sure if a list item applies to your research, read the appropriate section before selecting a response.

Materials & experimental systems

n/a	Involved in the study	
[ ]	[x]	Antibodies
[ ]	[x]	Eukaryotic cell lines
[x]	[ ]	Palaeontology and archaeology
[ ]	[x]	Animals and other organisms
[ ]	[x]	Human research participants
[x]	[ ]	Clinical data
[x]	[ ]	Dual use research of concern

Methods

n/a	Involved in the study	
[x]	[ ]	ChIP-seq
[ ]	[x]	Flow cytometry
[x]	[ ]	MRI-based neuroimaging

Antibodies

Antibodies used	Anti-hCD3 (Biolegend, Cat. No. 317326, clone OKT3, Lot B317658, 1 µg/ml) anti-hCD28 (Biolegend, Cat. No. 302934, clone CD28.2, Lot B318445, 1 µg/ml) anti-mCD3 (Cat. No. 100331, clone 145-2C11, Lot B206702, 30 ng/ml), anti-mCD28 (Cat. No. 102112, clone 37.51, Lot B212837, 30 ng/ml), mouse anti-GC1qR/p32 (Abcam , Cat. No. ab24733, clone 60.11, lot no. GR3208495-4 dilution 1:1000), rabbit anti-tubulin (Santa cruz, Cat. No. Sc53646, clone 10D8, lot no. K0515, dilution 1:1000), goat anti-mouse-HRP (Jackson ImmunoResearch Cat. No. 115-035-166, Lot 124784, Dilution:1:10,000), goat anti-rabbit HRP (Jackson ImmunoResearch Cat. No. 115-035-166, Lot 128223, Dilution:1:10,000) Anti-mouse CD3ε-APC (Biolegend, Cat No. 100311, clone 145-2C11, lot B291091, dilution 1:100). Anti-mouse CD8a-alexa 488 (Biolegend, Cat No. 100723, clone 53-6.7, lot B254526, dilution 1:200). Anti-mouse CD4-BV 785 (Biolegend, Cat No. 100453, clone GK1.5, lot B236697, dilution 1:100). Anti-mouse CD25-PE (Biolegend, Cat No. 101904, clone 3C7, lot B247733, dilution 1:50). Anti-mouse LAG3-PerCP-Cy5.5 (Biolegend, Cat No. 125211, clone C9B7W, lot B261544, dilution 1:200). Anti-mouse PD-1-PerCP-Cy5.5 (Biolegend, Cat No. 135208, clone 29F.1A12, lot B302661, dilution 1:200). Anti-human CD3ε-APC (Biolegend, Cat No. 300439, clone UCHT1, lot B278610, 5ul per test). Anti-human CD4-FITC (Biolegend, Cat No. 317408, clone OKT4, lot B234953, 5ul per test). Anti-human CD8a ef450 (Biolegend, Cat No. 48008842, clone RPA-TB, lot 1988089, 5ul per test). Anti-human CD25-FITC (Biolegend, Cat No. 302604, clone BC-96, lot B253407, 5ul per test). Anti human LAG3-APC (Biolegend, Cat No. 369212, clone 7H2C65, lot B294090, 5ul per test). Anti human CD45RA-APC (Biolegend, Cat No. 304112, clone HI100, lot B276540, dilution 1:200). Anti human CCR7-FITC APC (Biolegend, Cat No. 353216, clone G043H7, lot B194490, dilution 1:200). Anti human PD-1-FITC (Biolegend, Cat No. 621611, clone A17188B, dilution 1:200). Rabbit anti-FLAG (Cell Signaling, Cat. No. 14793, clone 145-2C11, Lot B206702, Dilution 1:400). Rabbit IgG Isotype control (Cell Signaling, Cat. No.3900, Dilution: same concentration as corresponding primary antibody). Anti-mouse/human C1QBP-PE (Santa Cruz, Cat. No. sc-23884, Clone 60.11, Lot L0403, Dilution: 20 ul per test). TruStain FcXTM-Fc blocker CD16/32 (Biolegend, Cat. No. 101320, clone 93, Lot no. B275367, dilution 1:100) PE-

	conjugated anti-IFN-g antibody (Biolegend, Cat. No. 505807, Clone XMG1.2, Lot no. B240650, dilution 1:100), rabbit anti-p32 polyclonal antibody (gift from Prof. Tambet Teesalu, stock 4.5 mg/ml, Dilution: 1:100), rabbit anti-vWF (Abcam, Cat. No. ab6994, Lot GR3180938-1, Dilution 1:100), goat anti-rabbit-Alexa 488 (Abcam, Cat No. ab150077, dilution 1:500), or goat anti-rabbit Cy3 (Jackson ImmunoResearch, Cat No 111-165-144, Lot 123834, dilution 1:400).
Validation	All antibodies used were validated for the specific application and species by the manufacturers and validation data and references were provided in the manufacturers website. All antibodies were used according to the manufactures instructions only for the validated species and application. Validation statements on the manufacturer's website for all primary antibodies used:C1QBP-PE conjugated antibody (Santa Cruz. Cat No. sc-23884) was tested by the manufacturer and have reported application for flow cytometry on murine and human cells.<br>Anti-FLAG (Cell Signaling , Cat No.14793) was tested by the manufacturer and have reported application for flow cytometry on murine and human cells. Anti-Tubulin (Santa cruz, Cat No.sc-53646) was tested by the manufacturer and have reported application for WB on murine and human cells. Anti-Von Willebrand Factor (vWF) antibody (Abcam, Cat No. ab6994) was tested by the manufacturer and have reported application for IHC-P on human cells and we have publications where we tested in mouse cells and tissue. Murine targeting Biolegend antibodies listed were tested by the manufacturer for flow cytometry on murine cells. Human targeting Biolegend antibodies listed were tested by the manufacturer for flow cytometry on human cells. Anti-human CD8a ef450 (Invitogene, Cat No. 48008842) was tested by the manufacturer and have reported application for flow cytometry on human cells. Anti-GC1qR/p32 (Abcam, Cat No. ab24733) was tested by the manufacturer and have reported application for WB on murine and human cells. Purified Rat Anti-Mouse CD31 (BD Biosciences, Cat No. 557355) was tested by the manufacturer and have reported application for flow cytometry on murine cells. Rabbit anti-p32 polyclonal antibody was provided by Prof. Tambet Teesalu who has publications testing the antibody in both murine and human tissue (IF, IHC)<br>Secondary antibodies:<br>Goat anti-Rabbit Alexa 647 (Abcam, Cat No. ab150079), Goat anti-mouse Alexa 647 (Abcam, Cat No. ab150115) and Goat anti-Rabbit AlexaFluor 488 (Abcam, Cat No. ab150077), were tested by the manufacturer for flow cytometry and IF.<br>Mouse anti Rat Alexa 647 (Biolegend, Cat No. 407511) was tested by the manufacturer for flow cytometry and IF.<br>Goat anti Rabbit Cy3 (Jackson ImmunoResearch, Cat No. 111-165-144) was tested by the manufacturer for flow cytometry and IF.<br>Goat anti Mouse HRP (Jackson ImmunoResearch, Cat No. 115-035-166) and Goat anti Rabbit HRP (Jackson ImmunoResearch, Cat No. 111-035-144) were tested by the manufacturer for WB.

Eukaryotic cell lines

Policy information about cell lines

Cell line source(s)	Human embryonic kidney 293T cells (HEK 293T), U87, U118, U178 were obtained from the American Type Culture Collection (ATCC, Manassas, VA, USA). U251 human GBM cell line was obtained from the European Collection of Authenticated Cell Cultures (ECACC) (Porton Down, Salisbury, UK). GBM83, GBM1005, GBM1027, GBM1079, GBM1051 were obtained from Prof. Ichiro Nakano. G179, G26, G7 were obtained from Prof. SM Pollard/Peter Dirks. BL line was obtained from Prof. Santosh Kesari. NCH421K line was obtained from Prof. C. Herold-Mende and received from Prof. Tambet Teesalu. Human primary cells H-6067, H-6034, H-6013, H-6044 were purchased from cell biologics, and N7805-100 was purchased from Gibco. 005 is a murine glioma stem cell line generated from a lentiviral HRasV12 induced tumor in a p53-/- knockout mouse and GBM83 is a patient tumor-derived GSC (newly diagnosed female, 72 years old, 30-35% Ki67 index). AFFR53 are transformed primary astrocytes with HRas-shp53 lentivirus. O1 cells were derived from FGFR1mut-shp53 lentiviral induced tumor. For a complete list and summary of all the cell lines please refer to Supplementary Table 1.
Authentication	Cell lines from ATCC have been thoroughly tested and authenticated; ATCC uses morphology, karyotyping, and PCR based approaches to confirm the identity of human cell lines. U251 cell line was authenticated by the European Collection of Authenticated Cell Cultures (ECACC) using morphology, karyotyping, PCR-based techniques, and Cytocrome oxidase I assay, following manufacturer validated procedures. Murine derived cells lines (005, AFFR53 and O1) and patient derived cell lines were not authenticated by the authors.
Mycoplasma contamination	All cell lines were routinely tested for mycoplasma using the EZ-PCR-Mycoplasma test kit (Biological Industries). All cells used were tested negative for mycoplasma contamination.
Commonly misidentified lines<br>(See ICLAC register)	The ICLAC identifies U118 as misidentified line, a derivative of U138, possibly sharing a common donor. We only used this cell line once in the manuscript to check expression of p32 on surface (FACS analysis).

Animals and other organisms

Policy information about studies involving animals; ARRIVE guidelines recommended for reporting animal research

Laboratory animals	All the animals were maintained within the Tel Aviv University specific pathogen free (SPF) facility. Groups of up to five mice per IVC cage (Lab-Products) were housed on a 12 h light/dark cycle, on autoclaved ASPEN wood chips bedding, at an ambient temperature of 22°C±1°C, with humidity controlled at 50%. The animals used for the in vivo experiments were randomized to receive either control or CART treatment. No statistical method was used to pre-determine sample size, but the sample size was chosen to be adequate to receive significant results as determined by preliminary experiments. Mice that died for unknown reasons (no tumor lesions were found) were excluded from analysis. C57Bl/6J and Nude male and female 8-12 weeks old mice were purchased from Envigo Jerusalem Israel.
Wild animals	The study did not involve wild animals.
Field-collected samples	The study did not involve field-collected samples

Ethics oversight	Animal experiments were approved by the animal care and use committee (IACUC) of Tel Aviv University (approval protocol no. 04-16-073) and conducted in accordance with NIH guidelines.

Note that full information on the approval of the study protocol must also be provided in the manuscript.

Human research participants

Policy information about studies involving human research participants

Population characteristics	Fresh surgical samples of GBMs were obtained from Tartu University Clinics, Tartu, Estonia under protocols approved by the Ethics Committee of the University of Tartu, Estonia (permit #243/T27).
Recruitment	Fresh surgical samples were collected and chosen according to patients' glioma grade upon pathology analysis.
Ethics oversight	All methods for the use of human samples were carried out in accordance with relevant guidelines and regulations. The collection and use of human samples was approved by the Research Ethics Committee of the University of Tartu, Estonia (permit #243/T-27). Freshly excised human samples were obtained during surgeries from Department of Neurosurgery, Tartu University Hospital, Estonia. The informed consent was obtained from all patients.<br>Human T cells from healthy donors were purchased from Magen David Adom (Blood services Center) in Israel. All experiments complied with protocols that were approved by Institutional Review Board at Tel Aviv University and Magen David Adom.

Note that full information on the approval of the study protocol must also be provided in the manuscript.

Flow Cytometry

Plots

Confirm that:

- [x] The axis labels state the marker and fluorochrome used (e.g. CD4-FITC).
- [x] The axis scales are clearly visible. Include numbers along axes only for bottom left plot of group (a 'group' is an analysis of identical markers).
- [x] All plots are contour plots with outliers or pseudocolor plots.
- [x] A numerical value for number of cells or percentage (with statistics) is provided.

Methodology

Sample preparation	Brain tumors (GFP+ tissue resected under fluorescent microscope) were dissociated using a Neural dissociation kit (Miltenyi Biotec) according to manufacturer's instructions and the resulting cell suspension was cleaned of debris (myelin) via Percoll (Sigma) density gradient centrifugation. CD45- cells were enriched using anti-mouse-CD45 magnetic-microbead and MS columns (Miltenyi Biotec) according to manufacturer's instructions.<br>For dissociation of lung tissue, after organ extraction, tissue was cut in small pieces and then incubated with collagenase type IV (Worthington #LS004186), Dispase II (Sigma-Aldrich, 4942078001) and DNAse I (Sigma-Aldrich, 11284932001) in a 37 C water bath for 40 minutes under constant stirring. The digested mixture was then strained and red blood cells were lysed prior to staining.<br>For flow cytometry analysis 100,000 cells were stained with the appropriated antibodies according to the antibodies manufacture instructions. Briefly, the cells were incubated with TruStain FcXTM-Fc blocker CD16/32 (Biolegend, Cat. No. 101320, clone 93, Lot no. B275367, dilution 1:100) for 15 min to reduce non-specific staining, followed by staining with primary antibodies listed above for 30 min at 4 ºC. Cells were washed and resuspended in PBS.
Instrument	Attune NxT Flow Cytometer
Software	Kaluza 2.1 software was used for all flow cytometry experiments.
Cell population abundance	No sorting was conducted
Gating strategy	Figures describing the gating strategy for selecting cells and singlets is provided in supplementary figure 9. First, density plots of FSC-A and SSC-A were generated and most of the population was gated with exclusion of dead cells and debris. Next, plots of SSC-A and SSC-H were generated for doublets discrimination and gating on singlets population. Boundaries between positive staining and negative staining was determined using an isotype control antibody for each staining.

- [x] Tick this box to confirm that a figure exemplifying the gating strategy is provided in the Supplementary Information.
